# Overview of Antagonists Used for Determining the Mechanisms of Action Employed by Potential Vasodilators with Their Suggested Signaling Pathways

**DOI:** 10.3390/molecules21040495

**Published:** 2016-04-15

**Authors:** Yean Chun Loh, Chu Shan Tan, Yung Sing Ch’ng, Mariam Ahmad, Mohd Zaini Asmawi, Mun Fei Yam

**Affiliations:** School of Pharmaceutical Sciences, Universiti Sains Malaysia, 11800 Minden, Penang, Malaysia; nicklesloh@hotmail.com (Y.C.L.); weazley90@hotmail.com (C.S.T.); yungsing1003@hotmail.com (Y.S.C.); mariam@usm.my (M.A.); amzaini@usm.my (M.Z.A.)

**Keywords:** vasodilators, antagonists, signaling pathway, blood vessel, vascular tone

## Abstract

This paper is a review on the types of antagonists and the signaling mechanism pathways that have been used to determine the mechanisms of action employed for vasodilation by test compounds. Thus, we exhaustively reviewed and analyzed reports related to this topic published in PubMed between the years of 2010 till 2015. The aim of this paperis to suggest the most appropriate type of antagonists that correspond to receptors that would be involved during the mechanistic studies, as well as the latest signaling pathways trends that are being studied in order to determine the route(s) that atest compound employs for inducing vasodilation. The methods to perform the mechanism studies were included. Fundamentally, the affinity, specificity and selectivity of the antagonists to their receptors or enzymes were clearly elaborated as well as the solubility and reversibility. All the signaling pathways on the mechanisms of action involved in the vascular tone regulation have been well described in previous review articles. However, the most appropriate antagonists that should be utilized have never been suggested and elaborated before, hence the reason for this review.

## 1. Introduction

Cardiovascular diseases are known to be the number one killer in the world, compared to other diseases such as disorders of the blood vessels and the heart. Hypertension is one of the main causes of cardiovascular diseases, and appropriately named as the silent killer due to it being an asymptomatic disease. In addition, it leads to a variety concomitant diseases, including stroke, heart diseases, kidney failure, cerebrovascular diseases, and more [1].Typically, hypertension has been categorized into four classes by the seventh report of Joint National Committee, which includes the normal and pre-hypertension stages, hypertension stage 1 and hypertension stage 2 [2]. Although there are many kinds of anti-hypertensive drugs present in the market nowadays, most have low effectiveness and undesired chronic side effects. Therefore, finding novel anti-hypertensive drugs is still a topic of huge interest to current researchers.

Hypertension is defined as having persistently high pressure exerted throughout the wall of blood vessels [3]. Blood vessels isolated from living organisms are commonly used for the *in vitro* studies on anti-hypertensive drugs researches [4]. Blood pressure has always been regulated in a narrow range to convey sufficient perfusion for tissues without causing any harm on the vascular system, especially the endothelium and vascular smooth muscle cells. Therefore, it is necessary to focus on understanding the signaling mechanism pathways involved in vascular tone regulation, including its way of signaling amplification by producing second messengers and the interaction between enzyme-linked, channel-linked, and G-protein coupled receptors. Plenty of reviews and research articles have discussed the probable signaling mechanism pathways involved in vascular tone regulation, providing great references for researchers, yet none of those discussed which of the signaling mechanisms pathway are significant, or suggest the most appropriate antagonists that should be utilized in respect to their corresponding receptors during studies of mechanistic pathways employed by the test compounds claimed to be exerting vasodilative effects. Therefore, the aim of this review is to provide a general view on the types of mechanistic pathways commonly employed by those researches that are related to this topic and the type of antagonists used. In addition, we aim to provide ideas on which antagonist is the most appropriate to use based on its specificity, selectivity, solubility, reversibility, and affinity to the receptors or enzymes. Finally, we aim to suggest the significance of signaling mechanism pathways to be employed.

This literature review focuses on the articles published during the latest five years (2010 to 2015) and abstracted in PubMed, strictly focusing on studies which meet at least two criteria: (a) the research must involve the use of blood vessels and (b) they must be studies of signaling pathway mechanism of action. Referring to these criteria, 257 articles were selected and reviewed in a comprehensive manner in order to meetthe aims of this review. We did not include in this review papers on reactive oxygen species and unrelated types of mechanistic studies.

## 2. Types of Blood Vessels

**Figure 1:**
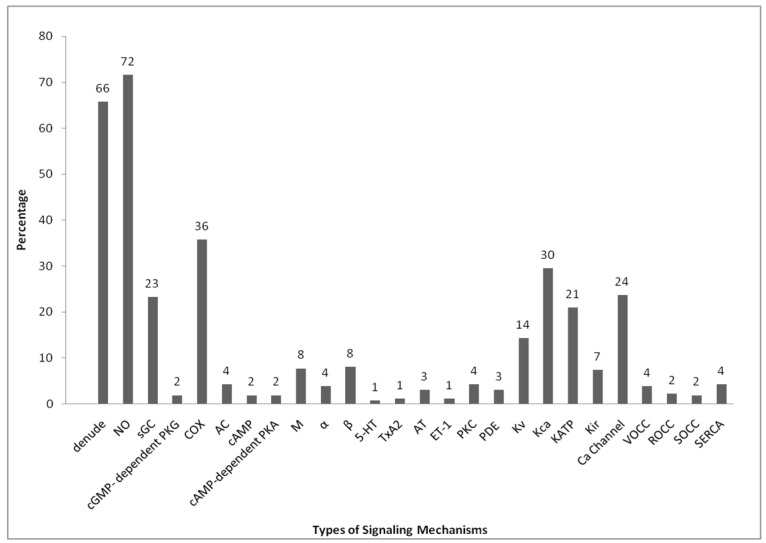
Percentage summary of signaling mechanism pathway studies conducted in research on blood vessel vasodilation published in PubMed from the year 2010 to 2015, as shown in Table 1. There were a total of 257 published research articles that performed research related to the vasodilation of aorta with studies on mechanisms of action within the specified period.

All studies involved a classical pharmacological approach using *in vitro* screening on isolated blood vessels, as shown in the Figure 1. A majority of the studies (67%) preferred the use of aorta rings for their *in vitro* studies on vasodilation, followed by mesenteric artery, coronary artery, pulmonary artery, renal artery, carotid artery, basilar artery, femoral artery, retinal arterioles, cerebral artery, and tail artery. Less than 1% of the studies were performed using saphenous vein, branchial artery, gonadal artery, internal mammary artery, caudal artery, prostatic small artery, afferent arterioles, gallbladder strips, ophthalmic artery, omental artery, bone resistance artery, splenic artery, iliac artery, umbilical artery, and gracilis arterioles.

The rat aorta modeling design is the most commonly utilized method for studies on vascular function as well as studies on cell signaling. Using this method, the orientation variation of the smooth muscle cells can be reduced and the damage to the intima surface of the vessel can be minimized during isolation and hanging of the aorta inside the organ bath. In addition, the rat aorta model is a cost-effective, fast, and simple way for *in vitro* and *in vivo* vasodilation studies. Hence, it is known to be the “golden tool” in pharmacological researches [5].

## 3. Signaling Mechanisms Involved in Vasodilation Studies

The primary pathways involved in the vasodilation of blood vessels such as NO and COX are the most commonly used in the mechanism studies, since both of these pathways are well-studied EDRFs. They are subsequently followed by the potassium channels, which can be subdivided into Kca, K_ATP_, Kv and Kir, channels. Calcium channels are essentially involved in the regulation of vascular tone, hence 24% of the researches in vasodilation studies were conducted on it. It is also because calcium channels can be divided into a few groups. Some of the researches that have been done were focused on those groups separately, such as VOCC, ROCC, SOCC, as well as the SERCA signaling pathways. The signaling pathway studies on enzyme-linked receptors such as sGC come after the calcium channels, while few researches had been done on AC. Other than those, a few other mechanisms such as PKG, cAMP, PKA, M, 5-HT, ET-1, TxA_2_, AT, PKC, PDE, and α- and β-adrenergic receptors have been included in these studies as well.

## 4. Endothelium-Derived Relaxing Factors (EDRFs)

In vascular tone regulation, both endothelium-derived relaxing factors (EDRFs) and endothelium-derived contracting factors (EDCFs) produce their effect via interaction between the endothelium and vascular smooth muscle cells. The most well-studied EDRFs are nitric oxide (NO), which is produced by endothelial nitric oxide synthases (eNOS), and prostacyclin (PGI_2_), produced by prostacyclin synthases within the endothelium [263,264,265,266,267].

### 4.1. Nitric Oxide (NO)

The highest percentage of the mechanism of actions summarized in Figure 1 belongs to the nitric oxide pathway at 72%, which are 184 out of 257 of the researches on this pathway. Nitric oxide is produced by the endothelial nitric oxide synthase (eNOS) in the endothelium from the breakdown of l-arginine. Therefore, to determine whether the relaxation induced by the extract is by this pathway, the specific inhibitor most commonly used is l-N^G^-NitroArginineMethyl Ester (l-NAME), followed by l-N^G^-NitroArginine (l-NNA). l-NNA is the first synthetic NOS inhibitor which acts competitively and selectively to eNOS [268]. However, l-NNA was claimed to bind with different isoforms. The binding of l-NNA to eNOS and nNOS is a time-dependent process with a relatively slow reversal [269,270]. l-NNA can show excellent stability in aqueous environment and has low toxicity, but due to its poor solubility at neutral pH, l-NAME is preferable, even though it is a weak NOS inhibitor [271,272]. The bioactivation of the l-NAME proceeds at a moderate rate in physiological buffers, but it is markedly accelerated in blood or the vascular endothelium [273]. Eventually, the percentage of relaxation caused by the extract will be reduced in the presence of eNOS inhibitor if the action of the active compound is via this pathway.

### 4.2. Prostacyclin (PGI_2_)

The second most frequently-studied mechanism of action is the prostacyclin (PGI_2_) signaling pathway (36%), which comprised 92 out of the 257 of the articles reviewed. PGI_2_ is produced by prostacyclin synthase from the intermediate prostaglandin H_2_, which is derived from AA and catalyzed by COX. Typically, COX can be classified into two types of isoforms. COX 1 is claimed to be responsible for the synthesis of prostaglandin and thromboxane, whereas the inducible COX 2 plays an important role in the synthesis of prostaglandin for the inflammatory cells as well as in the central nervous system [274,275]. From the literature, the studies on this pathway were commonly conducted using non-steroidal anti-inflammatory drugs (NSAIDs) as COX inhibitors. The most commonly used was indomethacin, followed by ibuprofen, meclofenamic acid, and diclofenac. Indomethacin is a potent inhibitor of both COX 1 and COX 2. However, there is an order of potency of the various inhibitors suggested, which is diclofenac > indomethacin > nimesulide ≈ meloxicam ≈ piroxicam [276]. Prostacyclin is one of the major relaxing factors derived from the endothelium. All the NSAIDs vary in their ability to inhibit both COX 1 and COX 2. Based on their mechanisms of inhibition, they can be classified into three broad categories. For instance, those that are able to bind reversibly and competitively to COX 1 and COX 2 are categorized in category 1. These include mefenamic acid, piroxicam, and ibuprofen. Those with rapid lower-affinity reversible binding, followed by the time-dependent with higher-affinity and slow reversible binding to both COX 1 and COX 2, form the second category, examples of which include diclofenac, flubiprofen, and indomethacin. Lastly, those with rapid reversible binding followed by covalent modification of both COXs, such as aspirin, are classified into category 3 [277,278]. Therefore, if the tested compound exhibits its vasodilator effect through the synthesis of prostacyclin, its percentage of relaxation will be significantly decreased in the presence of a COX inhibitor.

## 5. Enzyme-Linked NO pathway

### Soluble Guanylyl Cyclase (sGC)

In vascular tone regulation, the soluble type of guanylyl cyclase (sGC) is strictly related to the NO that diffuses into the adjacent VSMCs. Some of the researchers assumed that it is one of the pathways of NO/cGMP and 23% (60 out of 257) of the research involved the study of this mechanism. Two types of inhibitors were utilized to study these mechanisms, which are methylene blue (MB) and 1H-[1,2,4] oxadiazolo [4,3-*a*]quinoxalin-1-one (ODQ). MB was claimed to not be a true inhibitor of sGC. It can only act as a cGMP-lowering agent and partially prevents the nitrodilator-dependent activation of sGC by generating oxygen-free radicals. It has been described as a NO-release inhibitor [279] and does not enter the cells unless the membrane is permeabilized [280]. Therefore, MB is more appropriately used to determine the cGMP-dependent mechanism pathway since it is a non-selective inhibitor of sGC. However, the second type of inhibitor, ODQ, having an IC_50_ of around 20nM, was claimed to be a non-reversible but more selective inhibitor to the sGC enzyme [281]. ODQ can be solubilized by using DMSO and has been widely used to differentiate cGMP-mediated effects of NO from cGMP-independent effects [282,283]. ODQ apparently inhibits sGC through oxidation on the heme group of the enzyme. In this case, if the test compound exerts its relaxant effect through stimulation of the sGC activity, it will have a lower vasodilation effect on the isolated tissue in the presence of the ODQ inhibitor. Nonetheless, if the test compound’s relaxing effect is strictly dependent on the production of the cGMP, then the subsequent percentage of relaxation exerted on the isolated tissue will significantly decrease in the presence of methylene blue, or else it will remain unchanged in the presence of ODQ. A few studies (2% or 6 out of the 257 papers) included research on the protein kinase G (PKG) mechanism pathway. The most commonly used cGMP-dependent PKG inhibitor (cGKI) is Rp-8-Br-PET-cGMPs. It is a metabolically stable, competitive, reversible, and non-selective blocker since it was claimed to be able to block both PKG 1 and PKG 2 [284].

## 6. G-Protein-Coupled Receptors

G-protein-coupled receptor (GPCR) is a single polypeptide that has seven transmembrane-spanning α-helices. It will respond to ligands through the activation of the G-proteins, which are located on the intracellular surface of the cell membrane. In general, G-proteins can be categorized into α, β, and γ types. They are functionally switched on when bound to guanosine triphosphate (GTP) and will revert to resting state when they are bound to guanosine diphosphate (GDP) [285]. Once GTP is bound to the G-protein and activates it, the G-protein will be cleaved into Gα and Gβγ dimers. Typically, Gα-proteins can be classified into G_q_α, G_i_α, and G_s_α which are functionally responsible for different roles in signaling transduction of blood vessels. G_q_α-protein tends to activate PLC-β for increasing the production of the second messengers IP_3_ and DAG, whereas G_i_α is functionally opposed to G_q_α. Nonetheless, G_s_α-protein tends to activate adenylyl cyclase (AC) to increase the production of the cAMP second messenger, which is involved in the vasodilation pathway. In the endothelium, there are a few G_q_α-protein-coupled receptors such as angiotensin receptor (AT_2_) [286,287,288], serotonin (5-HT_1D_) receptor [289,290,291,292], bradykinin receptor (B_2_) [293,294], muscarinic receptor (M_3_), and endothelin receptor (ET_B_R) [295,296,297,298,299]. Whereas in the VSMC, the α_1_-adrenergic receptor [288,292], muscarinic receptor (M_3_) [300,301,302], angiotensin receptor (AT_1_) [286,303,304,305,306], endothelin receptor (ET_A_R & ET_B_R) [292,295,296,297,298,299,307], serotonin receptor (5-HT_2_) [289,290,292,308], and thromboxane receptor (TxA_2_) [288,292,309], are G_q_α-protein-coupled receptors and tend to produce a direct effect in the increase of intracellular concentration of calcium by passing through the PLC-dependent pathway.

### 6.1. β-Adrenergic Receptors

Activation of the G_s_α-protein-coupled receptors, such as the β_2_-adrenergic receptor and the IP receptor, will stimulate the activity of adenylyl cyclase (AC) in the VSMCs. These G_s_α-PCRs have a less dominant presence in the VSMCs compared to G_q_α-PCRs, hence less research were conducted on these mechanisms. Of the studies, only 4% (11 out of 257) were on the AC pathway, 2% (5 out of 257) on the cAMP, and 2% (5 out of 257) on the cAMP-dependent protein kinase A (PKA) pathway. Due to the PKA’s tetrameric composition in both catalytic and regulatory subunits, the inhibition of PKA can be achieved by blocking the ATP binding site or by inhibiting the analogue cAMP [310,311]. Generally, SQ22536 is more commonly utilized *in vitro* as an AC inhibitor, which is claimed to be more potent than MDL12330A. SQ22536 is specific to cAMP signaling and does not block the actions of other compounds that are not related to cAMP signaling [312]. SQ 22536 is cell-permeable and soluble in water. Other than that, Rp-cAMPs and H-89 have been used to study the cAMP and cAMP-dependent PKA mechanisms of action, respectively. Rp-cAMPs is a cell-permeable, reversible, competitive, and selective inhibitor for PKA. It is also resistant to the degradation of PDE, which makes it as an excellent tool to study these mechanisms [313]. H-89 is claimed to be a potent, cell-permeable, and reversible inhibitor of PKA, but it has been claimed that H-89 will exhibit unwanted effects such as influencing the ion currents and contraction of the smooth muscle [314] and activation of the PKG pathway [315]. Due to the unclear metabolic fate of H-89 and another inhibitor KT-5720, Rp-cAMPs is generally recommended for studies on this mechanism. In this case, if the test compound has employed this pathway for the regulation of the vascular tone, the expected outcome of the vasodilation effect would be decreased in the presence of this inhibitor. In regard to this pathway, the β_2_-adrenergic receptor, which is located on the VSMCs, actually employs this pathway for vasodilation as well. Only 8% (21 out of 257 studies) have focues onthis pathway. Even though its effect in the regulation of the vascular tone of blood vessels is not dominant, it is significant in myocytes. Propanolol is most widely used as the β-blocker in their studies. It is a non-selective blocker of the β-adrenergic receptor. Other than that, nadolol, atenolol, and pindolol have been used for these mechanistic studies as well. However, according to the classification of β-blockers, propanolol, nadolol, and pindolol are classified as non-selective β-blockers, whereas atenolol is more selective in cardio β_1_-blockers [316]. Propanolol is preferable for the screening of isolated tissue as compared to cardio-selective β-blockers. If the test compound involves this pathway, the resulting vasodilation effect will be decreased in the presence of β-blockers.

### 6.2. Muscarinic Receptors (M_3_)

There are five subgroups of muscarinic receptors, but only M_3_ receptors are present in the blood vessels. M_3_ receptors are G_q_α-protein-coupled receptors, predominant in the endothelial cells in which they mediate vasodilative effect through stimulating the cascade of signaling pathways within the endothelium. There are M_3_ receptor selective antagonists present in the market, such as *N*-methylatropine and tiotropium, but evidence proves that M_1_ receptors are actually present in the endothelium [317]. However, that information still remains insufficient. Therefore, the non-selective muscarinic receptor atropine has been widely used to study this pathway, comprising approximately 8% (20 out of 257) of the studies. Atropine is a well-known muscarinic subtype non-specific antagonist, which acts as a competitive inhibitor with acetylcholine at the postganglionic muscarinic sites [318,319]. When atropine is bound to the M_3_ receptor, the subsequent vasodilation exerted by the test compound would be decreased significantly.

## 7. Vasoconstriction-Dominated Receptors

In studies about the mechanism pathways employed by test compounds causing vasodilation on isolated tissue, the mechanism of actions predominantly responsible for causing vasodilation should be well-determined This is especially true for eNOS, PGI_2_, sGC pathways, as well as their messengers produced. During these studies, researchers usually prioritize on enzymes and receptors which predominantly cause vasodilation upon their activation, such as eNOS, PGI_2_, AC, sGC, muscarinic receptors, and β-adrenoreceptors. This is because the vasodilation effect of the test substances should be dominating the effect of the vasoconstriction if the test substances in fact have a mild agonist effect on the vasoconstrictor-predominant receptors, such as serotonin receptors, ET_A_R, ET_B_R, AT-1, PKC, α_1_-adrenoreceptors, and T_X_A_2_ receptors. For instance, if the test substances have employed these signaling mechanism pathways, eventually they will result in vasoconstriction. Nonetheless, the test substances could act as blockers of these vasoconstriction-predominated receptors, which will cause more vasodilation. However, many of these mechanistic pathways might be included and need to add their effects to exhibit a major vasodilation effect. Therefore, most researchers did not conduct this type of mechanistic pathway studies since most of the results of each separate signaling mechanism study could be insignificant due to their minor contribution on the overall effect. From the articles, to determine the mechanism of action of the test compound, the percentage of relaxation in the experimental group where the antagonists have been applied before pre-contraction must be compared with the control group. However, from the data tabulation in Table 1, few studies have been conducted on the mechanism of action of vasoconstriction-dominated receptors. Those receptors, such as α_1_-adrenergic receptor (4%), serotonin receptor (1%), thromboxane receptor (1%), angiotensin 2 receptor (3%), and endothelin receptor (1%), are functionally dominant in the VSMCs. All these receptors are G_q_α-protein-coupled receptors and tend to act in receptor-operated pathways to increase the calcium concentration in the cytosol when stimulated by their selective agonists. A selective blocker for α_1_-adrenergic receptor, prazosin, was the most commonly used test compound. There are two subtypes of endothelin receptors present in the VSMCs, BQ-123 and BQ-788, which have been used as selective blockers for ET_A_R and ET_B_R respectively. Besides, the angiotensin 2 type 1 receptor (AT-1) has been widely studied by using its specific antagonists, such as losartan and valsartan, whereas, the selective inhibitors for thromboxane receptor (TxA_2_) and serotonin (5-HT_2_) the compounds used were ridogrel and katanserin, respectively. Other than those, the protein kinase C (PKC) and PDE enzymatic signaling pathways have also been studied using their respective inhibitors. For instance, the selective inhibitors of PKC commonly used are GF 109203X, staurosporine, Go 6983, chelerythrine, and highly-selective cell-permeable BIM. PDE present in the VSCMCs are commonly classified as PDE 5 and PDE 3. About 3% of the papers involved studies on this pathway. Examples of selective inhibitors of PDE 5 are sildenafil, dipyridamole, zaprinast, and T-1032, while milrinone is a selective inhibitor for PDE 3. However, the non-selective PDE inhibitor papaverine has been utilized as well in their studies.

## 8. Channel-Linked Receptors

These receptors are also known as ligand-gated channels, whereby the channels respond by binding with chemical messengers and react by allowing the ions, such as calcium, potassium, sodium, chloride, or magnesium, to enter or leave the cell. Typically, the reaction of these receptors is regulated by the membrane potential.

### 8.1. Potassium Channels

#### 8.1.1. Calcium-Dependent Potassium Channels (Kca)

According to Table 1, thisis the 3rd most abundantly studied topic (30%, 76 out of 257 papers) in the reviewed research. Kca channels consist of small-conductance (SKca), intermediate-conductance (IKca), and big-conductance calcium-activated potassium channels (BKca). SKca and IKca channels are more abundantly expressed in the endothelium, but expressed poorly in the VSMCs, hence it has been claimed they are able to contribute to the EDHF signals and react in the adjacent VSMCs [320,321,322,323,324,325,326], whereas the BKca channels are expressed in virtually all VSMCs. According to the analysis, the majority of the studies on this pathway have been conducted using the non-selective Kca channel blocker, tetraethylammonium ion (TEA). Even though TEA can completely abolish the delayed potassium channels current without affecting the transient sodium current [327], it is a non-selective Kca channel blocker. Therefore, to make the Kca channel signaling pathway clearer, SKca, IKca, and BKca channel blockers should be used to examine the Kca channel pathway mechanisms. Due to this reason, some of the researches were done by using different selective channel blockers. Blockers such as apamin were used as the SKca channel blocker, TRAM-34 and clotrimazole as selective blockers for IKca, and iberiotoxin, UCL 1684, and paxilline as the selective blockers for BKca channels [323]. A few of the researches have used charybdotoxin as a non-selective blocker to determine both BKca and IKca channels. Specific determination of both IKca and SKca channels is necessary due to their contribution as EDHF within the endothelium. Besides, both of these channels are functionally associated with calcium and calmodulin binding [328], and are indirectly involved in the regulation of vascular tone through their participation in generating membrane potential. The activation of the BKca channels are indirectly mediated by many of the second messengers such as PKA, PKG, and the nitric oxide that diffuse across the VSMC membrane. BKca channels are voltage and calcium concentration-dependent potassium channels, hence indicating their essential roles in controlling the vascular tone excitability. The vasodilation effect will be decreased in the presence of the BKca inhibitor since there is a high depolarizing voltage created on the VSMC membrane that can induce more influx of calcium ions into the cytosol and cause muscle contraction.

#### 8.1.2. ATP-Sensitive Potassium Channels (K_ATP_)

The second most frequently used channel in vasodilation research was ATP-sensitive potassium channels (K_ATP_), which were studied in 21% (54 out of 257) of the reports. This channel opens when the intracellular ATP concentration falls below 1 mmol/L [329,330]. Glibenclamide, a selective K_ATP_ channels blocker, is the only antagonist utilized by those researches to determine thismechanism of action [331]. A few endothelium-derived substances, such as calcitonin-gene related protein (CGRP) and hydrogen sulfide (H_2_S), are able to stimulate K_ATP_ channels directly as well as indirectly mediated by cAMP-dependent PKA. The opening of these channels can cause membrane hyperpolarization, therefore the vasodilation effect will be decreased when the K_ATP_ channel antagonists are present [330].

#### 8.1.3. Voltage-Dependent Potassium Channels (Kv) and Inwardly Rectifier Potassium Channels (Kir)

There are two more potassium channels which are important based on their functional roles in the regulation of membrane potential, namely voltage-activated potassium channels (Kv) and inwardly rectifier potassium channels (Kir), which have been studied in 14% (37 out of 257) and 7% (19 out of 257) of the studies, respectively. They are less significant when compared to Kca channels and K_ATP_ channels because their functional roles are strictly regulated through the membrane potential. To determine the Kv channel pathway mechanisms, all the researchers used 4-aminopyridine (4-AP) as an antagonist and only one group used the Kv_7_ selective blocker to investigate this pathway. Kv channels consist of many different subgroups, therefore in order to inhibit these channels, a non-selective 4-AP blocker is preferable to using a selective inhibitor. It is an important component of the outward potassium conductance and is heavily regulated by kinases such as PKA, PKG and PKC, while sGC and NO tend to activate this channel directly [323]. These channels tend to provide the counterbalancing potassium efflux for the calcium influx through the voltage-operated calcium channel (VOCC) [332,333]. Therefore, if these channels were blocked, subsequent membrane depolarizing current will induce the calcium influx, resulting in more vasoconstriction. The Kir channels more readily induce the inward movement of potassium current and fasten the membrane potential to the resting stage [323]. Barium chloride (BaCl_2_) is the Kir channel blocker that has been most used so far according to the tabulated data, and will cause the decrease in relaxation response if the test compound employed this pathway. Barium chloride can selectively block Kir channels [334].

### 8.2. Calcium Channels

#### 8.2.1. Voltage-Operated Calcium Channels (VOCC)

Generally, there are three types of commonly-used selective L-type calcium channel blockers from the data obtained, which are the dihydropyridine class of nifedipine, the benzothiazepine class of diltiazem, and the phenylalkylamineclass of verapamil. These three classes of calcium channel blockers have different pharmacological effects due to the fact that they bind to different sites of the calcium channel. Diltiazem and verapamil have overlapping binding sites, whereas nifedipine binds to a distinct site. Therefore, the selectivity to the calcium channel blocker is arranged in ascending order as such nifedipine>diltiazem>verapamil, with the experiments executed using peripheral arterioles or coronary arteries [335]. However, verapamil is still the most widely-used calcium channel blocker in the studies. The dihydropyridines calcium channels blockers, such as nifedipine and nicardipine, have a higher vascular selectivity [336] and are the most smooth muscle selective class of the calcium channel blockers. From Table 1, 4% (10 out of 257) of the studies were conducted regarding this calcium channel blocker. Two types of methods were implemented in the researches to determine the VOCC mechanism pathway. One of the methods is that isolated tissue was incubated with the calcium channel antagonist before pre-contraction, and subsequently the cumulative concentration of the test compound was added. In this case, if the test compound has the potential to act as a VOCC opener in their mechanism of action in controlling the vascular tone in the control set of experiment, then the resulting vasodilation should be increased in the second set of the experiments where the VOCC antagonist was applied. By using this method, the inference of whether the test compound has employed this pathway as its mechanism of action can be made, but is not practically used because the action potential would abolish the significance of the result achieved. Therefore, the second method was the choice of the majority of researchers, as it is more suitable to determine whether their test compound can be a potential blocker for the VOCC. Isolated tissue is incubated and washed with calcium-free Krebs-Henseleit solution, which contains ethylenediamine tetraacetic acid (EDTA) or ethylene glycol tetraacetic acid (EGTA) with high potassium content (~50 mM). Both of them are chelating agents and appear as white crystalline powders, but EGTA is preferable because of its significantly higher affinity to the divalent calcium ions, while EDTA is preferable for divalent magnesium ions. The solution used to wash the isolated tissue should not contain any EGTA or EDTA. The tissue was then incubated with certain doses of the test compound for at least 10 min before adding cumulative doses of calcium chloride. The experimental group was compared to the control group (including the positive control) to determine the degree of significance. By using this method, if the contraction induced by the cumulative doses of calcium chloride is significantly decreased, this indicates that the test compound has potential to act as the antagonist of the VOCC.

#### 8.2.2. Store-Operated Calcium Channels (SOCC)

Regarding the intracellular release of calcium from the sacroplasmic reticulum stores, gandolinium (Gd^3+^) and 2-aminoethoxydiphenyl borate (2-APB) are most widely been used inhibitors to study this pathway. Around 2% of the researcher studied this pathway. 2-APB is an IP_3_R inhibitor and is able to inhibit the release of the intracellular stores of calcium from SR. It was claimed to be able to completely inhibit the release of calcium at high concentrations [337,338] and is also a reliable blocker of the SOCC, but an inconsistent inhibitor of IP_3_-induced calcium release [339]. Other than that, the Gd^3+^ is usually used together with the 2-APB as it is a selective blocker of the SOCC [340,341]. However, a few of the researchers were using non-competitive, selective, and cell-permeable SERCA blockers such as thapsigargin [342] to aid the calcium entry blocking effect from the SOCC into the SR during their studies on this mechanism of action. Like VOCC, two methods can be used to test this SOCC mechanism of action. The isolated tissue is pre-treated with the normal Krebs-Henseleit solution containing thapsigargin, Gd^3+^, as well as nifedipine to eliminate the VOCC effect for at least 10 min before the pre-contraction. Subsequently, cumulative doses of the test compound were added. If the test compound utilizes this pathway in its regulation of vascular tone, it will exhibit more relaxation than in the control group because the test compound can no longer induce the entry of calcium though the SOCC. However, another method is used to determine whether the calcium released from the sites of intracellular calcium store contributes to the auto-regulation of vascular tone. In this case, the isolated tissue was pre-treated with calcium-free Krebs-Henseleit solution containing the test compound for at least 20 min before the addition of phenylephrine. If there is a significant change in the percentage of contraction induced by PE, this indicates that the test compound plays a role in controlling the intracellular release of calcium into the cytosol.

## 9. List of Antagonists and Its Receptor

All the antagonists used in their respective signaling mechanism pathway studies as tabulated in Table 1 were accordingly recategorized in Table 2 to facilitate future researchers in related pharmacological research fields.

## 10. Conclusions

Understanding the microenvironment of blood vessel in regard to its regulation of vascular tone is essential for those who work in research to develop a new anti-hypertensive drug. Nonetheless, during the discovery of new drugs, researchers should be able to confirm and elaborate the most appropriate types of antagonists used according to their selectivity, specificity, affinity, reversibility and solubility to test on potential anti-hypertensive test compounds as well as the procedures used in order to obtain a clear and firm inference about the pathways that the test compound has employed for inducing the vasodilation effect. In this review paper, the essential signaling mechanism pathways that were most frequently demonstrated in anti-hypertensive research have been summarized, as well as the expected outcome of the studies of test compounds that involve a particular mechanism pathway. In the future, a general picture of the blood vessel microenvironment in vascular tone regulation, including signals of second messengers and the interaction among receptors and enzymes, should be used as a whole to provide a general view for the researchers who will work on this topic.

## Figures and Tables

**Table 1:** Data tabulation of the signaling mechanism pathways of vasodilation conducted in researches that were published in PubMed from the year 2010–2015.

TOV	D	NO	GC	PKG	COX	AC	cAMP	PKA	M	α	β	Sr	T	AT	ET	PKC	PDE	Kv	Kca	KATP	Kir	Ca	V	R	S	SE	Ref.
A	+	+	+	-	+	-	-	-	+	+	+	-	-	-	-	-	-	-	-	+	-	+	-	-	-	-	[6]
MA	+	+	+	-	+	+	-	-	-	-	-	-	-	-	-	-	-	+	+	+	+	+	-	-	-	-	[7]
A	+	+	+	-	-	-	-	-	-	+	-	-	-	-	-	-	-	-	+	-	-	+	-	-	-	-	[8]
A & SA	+	+	-	-	+	-	-	-	-	-	-	-	-	-	-	-	-	-	-	-	-	-	-	-	-	-	[9]
A	-	+	-	-	+	-	-	-	-	-	-	-	-	-	-	-	-	-	+	-	-	-	-	-	-	-	[10]
A	+	+	+	-	+	-	-	-	+	-	+	+	-	-	-	+	-	+	+	+	+	+	-	-	-	-	[11]
A	+	+	-	-	+	-	-	-	-	-	-	-	-	-	-	-	-	-	-	-	-	-	-	-	-	-	[12]
A	-	+	-	-	-	-	-	-	-	-	-	-	-	-	-	-	-	-	-	-	-	-	-	-	-	-	[13]
A	+	+	-	-	+	-	-	-	-	-	-	-	-	-	-	-	-	-	-	-	-	-	-	-	-	+	[14]
A	+	+	-	-	-	-	-	-	-	-	-	-	-	-	-	-	-	-	-	-	-	-	-	-	-	-	[15]
A	+	-	-	-	-	-	-	-	-	-	-	-	-	-	-	-	-	+	+	+	+	+	-	-	-	-	[16]
A	+	+	-	-	-	-	-	-	+	-	-	-	-	-	-	-	-	-	-	-	-	+	-	-	-	-	[17]
MA	+	+	+	-	+	+	+	-	-	-	-	-	-	-	-	-	-	-	-	-	-	-	-	-	-	-	[18]
A	+	+	+	-	-	-	-	-	-	-	-	-	-	-	-	-	-	-	-	-		+		-	-	-	[19]
A	-	-	-	-	-	-	-	-	-	-	-	-	-	-	-	-	-	-	-	-	-	-	-	-	-	-	[20]
A & MA	+	+	-	-	-	-	-	-	-	-	-	-	-	-	-	-	-	-	-	-	-	-	-	-	-	-	[21]
A	+	+	+	-	-	-	-	-	-	-	-	-	-	-	-	-	-	-	+	-	-	+	-	-	-	-	[22]
A	+	+	-	-	+	-	-	-	-	-	-	-	-	-	-	-	-	-	+	+	-	-	-	-	-	-	[23]
RAS	-	+	-	-	+	-	-	-	-	-	-	-	-	-	-	-	-	-	+	+	-	-	-	-	-	-	[24]
A	+	-	-	-	-	-	-	-	-	-	+	-	-	-	-	-	-	-	-	-	-	-	-	-	-	-	[25]
A	+	+	-	-	+	-	-	-	-	-	-	-	-	-	-	-	-	-	-	-	-	-	-	-	-	-	[26]
A	+	+	+	+	-	-	-	-	-	-	-	-	-	-	-	-	+	-	-	-	-	-	-	-	-	-	[27]
RAS	-	+	-	-	+	-	-	-	-	-	-	-	-	-	+	-	-	-	-	-	-	-	-	-	-	-	[28]
A	-	+	-	-	+	-	-	-	-	-	-	-	-	-	-	-	-	+	+	+	-	+	-	-	-	-	[29]
A	+	+	-	-	-	-	-	-	-	-	-	-	-	-	-	-	-	+	+	+	+	-	-	-	-	-	[30]
A	+	+	-	-	+	-	-	-	-	-	-	-	-	-	-	-	-	-	-	-	-	+	-	-	-	-	[31]
A	+	+	-	-	+	-	-	-	-	-	-	-	-	-	-	-	-	+	+	+	+	-	-	-	-	-	[32]
A	+	+	+	-	+	-	-	-	+	-	+	-	-	-	-	-	-	-	+	+	-	+	-	-	+	+	[33]
A	+	+	-	-	+	-	-	-	+	-	-	-	-	-	-	-	-	-	+	+	+	+	-	-	-	-	[34]
CA	+	-	+	-	-	+	-	-	-	-	-	-	-	-	-	-	-	+	+	+	+	+	-	-	-	-	[35]
A	+	+	+	-	+	-	+	-	-	-	-	-	-	-	-	-	-	-	-	-	-	+	-	-	-	-	[36]
A	+	+	+	-	-	-	-	-	-	-	-	-	-	-	-	-	-	-	-	-	-	+	-	-	-	-	[37]
GA, BLA & A	+	+	+	-	+	-	-	-	-	-	-	-	-	-	-	-	-	-	-	-	-	-	-	-	-	-	[38]
A	+	+	-	-	-	-	-	-	-	-	-	-	-	-	-	-	-	-	-	-	-	+	-	-	-	-	[39]
CA	-	+	+	-	-	-	-	-	-	-	-	-	-	-	-	-	-	-	-	-	-	-	-	-	-	-	[40]
A	+	+	-	-	-	-	-	-	-	-	-	-	-	-	-	+	-	-	-	-	-	-	+	-	-	-	[41]
A	+	+	-	-	-	-	-	-	-	-	-	-	-	+	-	-	-	-	-	-	-	-	-	-	-	-	[42]
MA	+	-	+	-	-	-	-	-	-	-	-	-	-	-	-	-	-	+	+	+	-	-	-	-	-	-	[43]
A	+	+	-	-	+	-	-	-	-	-	-	-	-	-	-	-	-	-	-	-	-	-	-	-	-	-	[44]
A	-	-	+	-	-	-	-	-	-	-	-	-	-	-	-	-	+	-	+	-	-	-	-	-	-	+	[45]
A	-	+	-	-	+	-	-	-	+	-	-	-	-	-	-	-	-	+	+	+	-	-	-	-	-	-	[46]
A	+	+	+	-	-	-	-	-	-	-	-	-	-	-	-	-	-	+	-	-	-	-	-	-	-	-	[47]
A	-	+	-	-	-	-	-	-	-	-	-	-	-	-	-	-	-	-	-	-	-	+	-	-	-	-	[48]
A	+	+	-	-	-	-	-	-	-	-	-	-	-	-	-	-	-	-	-	+	-	-	-	-	-	-	[49]
MA	-	+	-	-	+	-	-	-	-	-	-	-	-	-	-	-	-	-	-	-	-	-	-	-	-	-	[50]
A	+	+	-	-	-	-	-	-	+	-	-	-	-	-	-	-	-	-	-	-	-	-	-	-	-	-	[51]
A	-	+	-	-	+	-	-	-	-	-	-	-	-	-	-	-	-	-	-	-	-	-	-	-	-	-	[52]
A	+	-	-	-	-	-	-	-	-	+	-	-	-	+	-	-	-	-	-	-	-	-	-	-	-	-	[53]
IMA	-	+	+	-	+	-	-	-	+	-	-	-	-	-	+	-	-	+	+	+	+	-	-	-	-	-	[54]
A	+	+	-	-	-	-	-	-	-	+	+	-	-	-	+	-	-	-	-	-	-	-	-	-	-	-	[55]
A	+	+	-	-	-	-	-	-	-	-	-	-	-	-	-	+	-	-	-	-	-	-	-	-	-	-	[56]
IMA	+	-	-	-	-	-	-	-	-	-	-	-	-	-	-	-	-	+	+	+	-	+	-	-	-	+	[57]
A	+	+	-	-	+	-	-	-	-	-	-	-	-	-	-	+	-	-	-	-	+	+	-	-	-	-	[58]
A	+	+	-	-	-	-	+	-	-	-	-	-	-	-	-	-	-	-	-	-	-	-	-	-	-	-	[59]
A	+	+	-	-	-	-	-	-	-	-	-	-	-	-	-	-	-	-	+	-	-	-	-	-	-	-	[60]
A	+	+	+	-	+	-	-	-	+	-	-	-	-	-	-	-	-	-	-	+	-	-	-	-	-	-	[61]
CBA	-	-	-	-	-	-	-	-	-	-	-	-	-	-	-	-	-	-	-	-	-	+	-	-	-	-	[62]
A	+	+	+	-	+	-	-	-	-	-	+	-	-	-	-	-	-	-	-	-	-	-	-	-	-	-	[63]
A	-	+	-	-	+	-	-	-	-	-	-	-	-	-	-	-	-	-	-	-	-	-	-	-	-	-	[64]
A	+	+	-	-	-	-	-	-	-	-	+	-	-	-	-	-	-	-	+	+	-	-	-	-	-	-	[65]
A	+	+	-	-	+	-	-	-	-	-	-	-	-	-	-	-	-	-	-	-	-	-	-	-	-	-	[66]
MA	+	+	-	-	-	-	-	-	-	-	-	-	-	-	-	+	-	-	-	-	-	-	-	-	-	-	[67]
A	+	+	+	-	+	-	-	-	-	-	-	-	-	-	-	-	-	+	+	+	+	+	-	-	-	-	[68]
A	+	+	+	-	+	-	-	-	-	-	+	-	-	-	-	-	-	-	+	+	+	+	-	-	-	-	[69]
A	+	+	-	-	-	-	-	-	-	-	-	-	-	+	-	-	-	-	-	-	-	-	-	-	-	-	[70]
CA	-	+	+	+	-	+	-	-	-	-	-	-	-	-	-	-	-	-	-	-	-	-	-	-	-	-	[71]
A	-	+	-	-	-	-	-	-	-	-	-	-	-	-	-	-	-	-	-	-	-	-	-	-	-	-	[72]
A	+	+	-	-	+	-	-	-	+	-	+	-	-	-	-	-	-	-	+	+	-	-	-	-	-	-	[73]
A	+	-	-	-	-	-	-	-	-	-	-	-	-	-	-	-	-	-	-	-	-	-	-	-	-	-	[74]
A	+	+	-	-	-	-	-	-	-	-	+	-	-	-	-	-	-	-	-	-	-	-	-	-	-	-	[75]
A	+	+	-	-	-	-	-	-	-	-	-	-	-	-	-	-	-	-	-	-	-	-	-	-	-	-	[76]
RA	-	+	-	-	-	-	-	-	-	-	-	-	-	-	-	-	-	-	+	+	+	-	-	-	-	-	[77]
A	+	+	-	-	-	-	-	-	-	-	-	-	-	-	-	-	-	-	-	-	-	-	-	-	-	-	[78]
A	+	+	-	-	+	-	-	-	-	-	-	-	+	-	-	-	-	-	-	-	-	-	-	-	-	-	[79]
A	-	+	-	-	-	-	-	-	-	-	-	-	-	-	-	-	-	-	-	-	-	-	-	-	-	-	[80]
A	-	+	-	-	-	-	-	-	-	+	+	-	-	-	-	-	-	-	-	-	-	-	-	-	-	-	[81]
A	+	+	+	-	+	-	-	-	+	-	+	-	-	-	-	-	-	-	+	+	-	-	-	-	-	-	[82]
MA	-	+	-	-	-	-	-	-	-	-	-	-	-	-	-	-	-	-	+	+	+	-	-	-	-	-	[83]
A	+	+	-	-	+	-	-	-	-	-	-	-	+	+	-	-	-	-	-	-	-	-	-	-	-	-	[84]
A	-	+	+	-	-	-	-	-	-	-	-	-	-	-	-	-	-	-	+	+	+	+	-	-	-	-	[85]
A	+	-	-	-	+	-	-	-	-	-	-	-	+	-	-	-	-	-	-	-	-	-	-	-	-	-	[86]
A	+	+	+	-	-	-	-	-	-	-	-	-	-	-	-	-	-	-	-	-	-	-	-	-	-	-	[87]
A	-	+	-	-	-	-	-	-	-	-	-	-	-	-	-	-	-	-	-	-	-	-	-	-	-	-	[88]
A	+	+	+	-	+	-	-	+	-	-	-	-	-	-	-	-	-	+	+	+	-	-	-	-	-	-	[89]
A	-	+	-	-	+	-	-	-	-	-	-	-	-	-	-	-	-	+	+	+	-	-	-	-	-	-	[90]
A	+	+	+	-	+	-	-	-	+	-	-	-	-	-	-	-	-	-	-	-	-	-	-	-	-	-	[91]
A	+	+	+	-	+	-	-	-	-	-	-	-	-	-	-	-	-	-	-	-	-	-	-	-	-	-	[92]
A	-	+	-	-	+	-	-	-	-	-	-	-	-	-	-	-	-	-	-	-	-	-	-	-	-	-	[93]
A	+	+	+	-	+	-	-	-	+	-	+	-	-	-	-	-	-	-	+	+	-	+	-	-	+	+	[94]
A	+	+	+	-	+	-	-	-	+	-	+	-	-	-	-	-	-	-	+	+	-	+	-	-	+	+	[95]
CRA	+	+	+	-	+	-	-	-	-	-	-	-	-	-	-	-	-	+	+	+	-	+	-	-	+	+	[96]
A	+	+	+	-	+	-	-	-	-	-	-	-	-	-	-	-	-	-	-	-	-	-	-	-	-	-	[97]
A	+	-	+	-	-	+	-	-	-	-	-	-	-	-	-	-	-	+	+	+	-	-	-	-	-	+	[98]
A	+	-	-	-	-	-	-	-	-	-	-	-	-	-	-	-	-	+	+	+	-	+	-	-	-	-	[99]
A	+	-	-	-	-	-	-	-	-	-	-	-	-	-	-	-	-	-	-	-	-	+	-	-	-	-	[100]
A	-	+	-	-	-	-	+	+	-	-	-	-	-	-	-	-	-	-	-	-	-	-	-	-	-	-	[101]
BA	+	-	-	-	-	-	-	-	-	-	-	-	-	-	-	-	-	+	+	+	+	+	-	-	-	-	[102]
A	+	+	+	-	+	-	-	-	+	-	+	-	-	-	-	-	-	+	+	+	-	+	+	+	-	-	[103]
A	+	-	-	-	-	-	-	-	-	-	-	-	-	-	-	-	-	-	-	-	-	+	-	-	-	-	[104]
A	+	+	-	-	-	-	-	-	-	-	-	-	-	-	-	-	-	-	-	-	-	-	-	-	-	-	[105]
A	-	-	-	-	-	-	-	-	-	-	-	-	-	+	-	-	-	-	-	-	-	-	-	-	-	-	[106]
A	+	+	+	-	+	-	-	-	-	-	+	-	-	-	-	+	-	+	+	+	-	+	-	-	-	-	[107]
A	+	-	-	-	-	-	-	-	-	-	-	-	-	-	-	-	-	+	+	+	-	+	+	+	-	+	[108]
A	+	+	+	+	+	-	-	-	+	-	-	-	-	-	-	-	-	-	+	-	-	-	-	-	-	-	[109]
A	+	+	-	-	+	-	-	-	-	-	-	-	-	-	-	-	-	-	+	-	-	+	-	-	-	-	[110]
A	+	+	-	-	-	-	-	-	-	-	-	-	-	-	-	-	-	-	-	-	-	-	-	-	-	-	[111]
A	+	+	-	-	-	-	-	-	-	-	-	-	-	-	-	-	-	-	-	-	-	+	-	-	-	-	[112]
A	+	+	-	-	+	-	-	-	-	-	-	-	-	-	-	-	-	-	-	-	-	-	-	-	-	-	[113]
CA	+	+	+	-	+	-	-	-	-	-	-	-	-	-	-	-	-	-	+	-	-	-	-	-	-	-	[114]
A	+	-	-	-	-	-	-	-	-	-	-	-	-	-	-	-	-	-	-	-	-	-	-	-	-	-	[115]
RA & A	-	+	-	-	-	-	-	-	-	-	-	-	-	-	-	-	-	-	-	-	-	-	-	-	-	-	[116]
A	+	+	-	-	+	-	-	-	-	-	-	-	-	-	-	-	-	-	-	-	-	+	-	-	-	-	[117]
RAS	+	-	-	-	-	-	-	-	-	-	-	-	-	-	-	+	-	-	-	-	-	+	+	-	-	-	[118]
A	+	+	-	-	+	-	-	-	+	-	-	+	-	-	-	-	-	-	-	-	-	-	-	-	-	-	[119]
A	+	+	-	-	-	-	-	-	-	-	-	-	-	-	-	-	-	-	-	-	-	-	-	-	-	-	[120]
A	+	-	-	-	-	-	-	-	-	-	-	-	-	-	-	-	-	-	-	-	-	-	-	-	-	-	[121]
A & TA	+	+	-	-	-	-	-	-	-	-	-	-	-	-	-	-	-	-	-	-	-	-	-	-	-	-	[122]
PA	-	-	-	-	-	-	-	-	-	-	-	-	-	-	-	-	-	-	-	-	-	+	-	-	-	-	[123]
A	+	+	-	-	-	-	-	-	-	-	-	-	-	-	-	-	-	-	-	+	-	-	-	-	-	-	[124]
A	-	+	-	-	-	-	-	-	-	-	-	-	-	-	-	-	-	-	-	-	-	-	-	-	-	-	[125]
FA	+	+	-	-	-	-	-	-	-	-	-	-	-	-	-	-	-	-	-	-	-	-	-	-	-	-	[126]
MA & A	+	+	+	-	-	-	-	-	-	-	-	-	-	-	-	-	-	+	+	+	-	-	-	-	-	-	[127]
CA	+	+	-	-	-	-	-	-	-	-	-	-	-	+	-	-	-	-	-	-	-	-	-	-	-	-	[128]
CA & SPA	+	+	-	-	-	-	-	-	-	-	-	-	-	-	-	-	+	-	+	-	-	+	-	-	-	-	[129]
CRA	+	+	-	-	+	-	-	-	-	-	-	-	-	-	-	-	-	+	+	+	-	-	-	-	-	-	[130]
A	+	-	-	-	-	-	-	-	-	-	-	-	-	-	-	-	-	-	-	-	-	-	-	-	-	-	[131]
MA	-	+	-	-	+	-	-	-	-	-	-	-	-	-	-	-	-	-	-	-	-	-	-	-	-	-	[132]
A	-	-	-	-	-	-	-	-	-	+	-	-	-	-	-	-	-	-	-	-	-	-	-	-	-	-	[133]
A	+	+	+	-	+	-	-	-	+	-	+	-	-	-	-	-	-	-	+	+	-	+	-	-	+	+	[134]
A	-	+	-	-	-	-	-	-	-	-	-	-	-	-	-	-	-	-	-	-	-	-	-	-	-	-	[135]
A	+	-	-	-	-	-	-	-	-	-	-	-	-	-	-	-	-	+	+	+	-	-	-	-	-	-	[136]
PA	+	+	+	-	+	-	-	-	-	-	-	-	-	-	-	-	-	-	+	-	-	-	-	-	-	-	[137]
PA	+	-	-	-	-	-	-	-	-	-	-	-	-	-	-	-	-	-	+	-	-	-	-	-	-	-	[138]
BA	-	+	-	-	+	-	-	-	-	-	+	-	-	-	-	-	-	-	-	-	-	+	-	-	-	-	[139]
A	+	-	-	-	-	-	-	-	-	-	-	-	-	-	-	-	-	-	-	-	-	-	-	-	-	-	[140]
A	+	-	-	-	-	-	-	-	-	-	-	-	-	-	-	-	-	-	-	-	-	-	-	-	-	-	[141]
A	-	-	-	-	-	-	-	-	-	-	-	-	-	-	-	-	-	-	-	-	-	-	-	-	-	-	[142]
A	+	-	-	-	-	-	-	-	-	-	-	-	-	-	-	-	-	-	-	-	-	-	-	-	-	-	[143]
IA	-	-	-	-	+	-	-	-	-	-	-	-	-	-	-	-	-	-	-	-	-	-	-	-	-	-	[144]
A	+	+	-	-	-	-	-	-	-	-	-	-	-	-	-	-	-	-	-	-	-	-	-	-	-	-	[145]
CA	+	-	-	-	-	-	-	-	-	-	-	-	-	-	-	-	-	-	-	-	-	-	-	-	-	-	[146]
A	+	-	-	-	+	-	-	-	-	-	-	-	-	-	-	-	-	-	-	-	-	-	-	-	-	-	[147]
A	+	-	-	-	-	-	-	-	-	-	-	-	-	-	-	-	-	-	-	-	-	-	-	-	-	-	[148]
A	+	-	-	-	-	-	-	-	-	-	-	-	-	-	-	-	-	-	-	-	-	-	-	-	-	-	[149]
A	+	-	-	-	-	-	-	-	-	-	-	-	-	-	-	-	-	-	-	-	-	-	-	-	-	-	[150]
RA	-	+	-	-	-	-	-	-	-	-	-	-	-	-	-	-	-	-	-	-	-	-	-	-	-	-	[151]
VA	-	+	+	-	-	+	-	-	-	-	-	-	-	-	-	-	-	-	-	-	-	-	-	-	-	-	[152]
A	+	+	-	-	-	-	-	-	-	-	-	-	-	-	-	-	-	+	+	-	-	-	-	-	-	-	[153]
A	+	+	-	-	-	-	-	-	-	-	-	-	-	-	-	-	-	+	+	-	-	-	-	-	-	-	[154]
A	+	-	-	-	-	+	-	-	-	-	-	-	-	-	-	-	+	-	-	-	-	-	-	-	-	-	[155]
GCA	-	+	-	-	-	-	-	-	-	-	-	-	-	-	-	-	-	-	-	-	-	-	-	-	-	-	[156]
PA	+	-	-	-	-	-	-	-	-	-	-	-	-	-	-	-	-	-	+	-	-	-	-	-	-	-	[157]
A	-	+	-	-	-	-	-	-	-	-	-	-	-	-	-	-	-	-	-	-	-	-	-	-	-	-	[158]
A	+	+	+	-	+	+	-	-	-	-	-	-	-	-	-	+	-	-	+	+	-	+	-	-	-	-	[159]
A	+	+	-	-	-	-	-	-	-	-	-	-	-	-	-	-	-	+	+	-	+	-	-	-	-	-	[160]
A	-	+	-	-	-	-	-	-	-	-	-	-	-	-	-	-	-	-	-	-	-	-	-	-	-	-	[161]
A	-	+	-	-	-	-	-	-	-	-	-	-	-	-	-	-	-	-	-	-	-	-	-	-	-	-	[162]
A	+	+	-	-	+	-	-	-	-	-	-	-	-	-	-	-	-	-	-	-	-	-	-	-	-	-	[163]
MA	-	+	-	-	+	+	-	+	-	-	+	-	-	-	-	-	-	-	+	+	-	-	-	-	-	-	[164]
CA	-	+	-	-	-	-	-	-	-	-	-	-	-	-	-	-	-	-	-	-	-	-	-	-	-	-	[165]
A	+	+	+	-	+	-	-	-	-	-	-	-	-	-	-	-	-	-	-	-	-	-	-	-	-	-	[166]
A	+	+	-	-	-	-	-	-	-	-	-	-	-	-	-	-	-	-	-	-	-	-	-	-	-	-	[167]
GCA	-	+	-	-	-	-	-	-	-	-	-	-	-	-	-	-	-	-	-	-	-	-	-	-	-	-	[168]
A	+	+	-	-	-	-	-	-	-	-	-	-	-	+	-	-	-	-	-	-	-	-	-	-	-	-	[169]
A	-	-	-	-	-	-	-	-	-	-	-	-	-	-	-	-	-	-	-	-	-	-	-	-	-	-	[170]
PA	-	-	-	-	-	-	-	-	-	-	-	-	-	-	-	-	-	-	-	-	-	-	-	-	-	-	[171]
A	-	-	-	-	-	-	-	-	-	-	-	-	-	-	-	-	-	-	-	-	-	-	-	-	-	-	[172]
CA	-	-	-	-	-	-	-	-	-	+	-	-	-	-	-	-	-	-	-	-	-	+	-	-	-	-	[173]
MA	+	+	-	-	-	-	-	-	-	-	-	-	-	-	-	-	-	-	-	-	-	-	-	-	-	-	[174]
A	-	+	-	-	+	-	-	-	-	-	-	-	-	-	-	-	-	-	-	-	-	-	-	-	-	-	[175]
A	+	+	+	-	+	-	-	-	-	-	-	-	-	-	-	-	-	-	-	-	-	+	-	-	-	-	[176]
CA	-	-	-	-	-	-	-	-	-	-	-	-	-	-	-	-	-	-	-	-	-	+	-	-	-	-	[177]
A	+	-	+	-	+	-	-	-	-	-	-	-	-	-	-	-	-	-	-	-	-	-	-	-	-	-	[178]
A	+	+	-	-	-	-	-	-	-	-	-	-	-	-	-	-	-	-	+	-	-	+	+	+	-	-	[179]
A	+	+	-	-	-	-	-	-	-	-	-	-	-	-	-	-	-	-	+	-	-	+	+	+	-	-	[180]
A	-	+	-	-	+	-	-	-	-	-	-	-	-	-	-	-	-	-	-	-	-	-	-	-	-	-	[181]
FA	+	+	-	-	+	-	-	-	-	-	-	-	-	+	-	-	-	-	-	-	-	-	-	-	-	-	[182]
A	+	+	-	-	-	-	-	-	-	-	-	-	-	-	-	-	-	-	-	+	-	-	-	-	-	-	[183]
CA	+	+	-	-	+	-	-	-	-	-	-	-	-	-	-	-	-	-	+	-	-	+	-	-	-	-	[184]
A	-	-	+	-	-	-	-	-	-	-	-	-	-	-	-	-	-	-	-	-	-	-	-	-	-	-	[185]
A	-	+	-	-	+	-	-	-	-	-	-	-	-	-	-	-	-	-	-	-	-	-	-	-	-	-	[186]
A	+	-	+	-	-	-	-	-	-	-	-	-	-	-	-	-	+	-	-	-	-	-	-	-	-	-	[187]
PA	-	+	-	-	-	-	-	-	-	-	-	-	-	-	-	-	-	-	-	-	-	-	-	-	-	-	[188]
A	+	-	-	-	-	-	-	-	-	+	-	-	-	-	-	-	-	-	-	-	-	-	-	-	-	-	[189]
MA	+	+	+	-	+	-	-	-	+	-	-	-	-	-	-	-	-	-	+	-	-	+	-	-	-	-	[190]
A	+	+	-	-	-	-	-	-	-	-	-	-	-	-	-	-	-	-	-	-	-	-	-	-	-	-	[191]
A	-	-	-	-	-	-	-	-	-	+	-	-	-	-	-	-	-	-	-	-	-	-	-	-	-	-	[192]
PA	+	+	-	-	+	-	-	-	-	-	-	-	-	-	-	-	-	+	+	+	+	+	-	-	-	-	[193]
A	+	+	-	-	-	-	-	-	-	-	-	-	-	-	-	-	-	-	-	-	-	-	-	-	-	-	[194]
A	-	+	-	-	-	-	-	-	-	-	-	-	-	-	-	-	-	-	-	-	-	-	-	-	-	-	[195]
A	+	+	-	-	+	-	-	-	-	-	-	-	-	-	-	-	-	-	-	-	-	-	-	-	-	-	[196]
PA	-	+	-	-	+	-	-	-	-	-	-	-	-	-	-	-	-	-	-	-	-	-	-	-	-	-	[197]
BA	+	-	-	-	-	-	-	-	-	-	-	-	-	-	-	-	-	-	+	-	-	-	-	-	-	-	[198]
PA	+	+	-	-	-	-	-	-	-	-	-	-	-	-	-	-	-	-	-	-	-	+	-	-	-	-	[199]
BA	-	+	-	-	+	-	-	-	-	-	-	-	-	-	-	-	-	-	+	-	+	-	-	-	-	-	[200]
PSA	+	+	+	-	+	-	-	-	-	-	-	-	-	-	-	-	-	+	+	+	-	+	-	-	-	-	[201]
A	+	+	+	-	+	-	-	-	-	-	-	-	-	-	-	-	-	+	+	+	-	-	-	-	-	-	[202]
MA	+	-	+	-	-	-	-	-	-	-	-	-	-	-	-	-	-	+	+	+	-	-	-	-	-	-	[203]
CA & FA	+	-	-	-	-	-	-	-	-	-	-	-	-	-	-	-	-	-	-	-	-	-	-	-	-	-	[204]
A	+	-	-	-	-	-	-	-	-	-	-	-	-	-	-	-	-	-	+	-	-	+	+	+	-	-	[205]
RA	+	+	-	-	+	-	-	-	-	-	-	-	-	-	-	-	-	+	+	+	-	-	-	-	-	-	[206]
AA	-	+	-	-	-	-	-	-	-	-	-	-	-	-	-	-	-	-	-	-	-	-	-	-	-	-	[207]
A	-	-	-	-	+	-	-	-	-	-	-	-	-	-	-	-	-	-	-	-	-	-	-	-	-	-	[208]
A	-	+	-	-	+	-	-	-	-	-	-	-	-	-	-	-	-	-	-	-	-	-	-	-	-	-	[209]
TA	-	-	-	-	-	-	-	-	-	-	-	-	-	-	-	-	-	-	-	-	-	-	-	-	-	-	[210]
A	+	-	-	-	-	-	-	-	-	-	-	-	-	-	-	-	-	-	-	-	-	-	-	-	-	-	[211]
A	+	+	-	-	-	-	-	+	-	-	-	-	-	-	-	+	-		-	+	-	-	-	-	-	-	[212]
A	+	-	-	-	-	-	-	-	-	-	-	-	-	-	-	-	+	-	-	-	-	+	-	-	-	-	[213]
A	+	+	-	-	-	-	-	-	-	-	-	-	-	-	-	-	-	+	+	-	-	+	+	+	-	-	[214]
PA	-	-	-	-	+	-	-	-	-	-	-	-	-	-	-	-	+	-	-	-	-	-	-	-	-	-	[215]
RAS	+	+	+	-	+	-	-	-	-	-	-	-	-	-	-	-	-	+	+	+	-	-	-	-	-	-	[216]
MA	-	-	-	-	+	-	-	-	-	-	+	-	-	-	-	-	-	-	-	-	-	-	-	-	-	-	[217]
CA	+	+	-	-	-	-	-	-	-	-	-	-	-	-	-	-	-	-	-	-	-	-	-	-	-	-	[218]
A	-	+	-	-	-	-	-	-	-	-	-	-	-	-	-	-	-	-	-	-	-	+	-	-	-	+	[219]
A	+	-	-	-	-	-	-	-	-	-	-	-	-	-	-	-	-	-	-	-	-	-	-	-	-	-	[220]
A	-	-	+	-	-	-	-	-	-	-	-	-	-	-	-	-	-	-	-	-	-	-	-	-	-	-	[221]
A	+	+	-	-	-	-	-	-	-	-	-	-	-	-	-	-	-	-	-	-	-	-	-	-	-	-	[222]
MA	-	+	+	+	+	+	+	-	-	-	-	-	-	-	-	-	-	-	+	-	-	-	+	-	-	-	[223]
CRA	+	+	-	-	+	-	-	-	-	-	-	-	-	-	-	-	-	-	+	-	-	+	-	-	-	-	[224]
A	+	+	-	-	-	-	-	-	-	-	-	-	-	-	-	-	-	-	-	-	-	-	-	-	-	-	[225]
A, CA, MA & RA	-	+	-	-	-	-	-	-	-	-	-	-	-	-	-	-	-	-	-	-	-	+	-	-	-	-	[226]
A	+	+	-	-	-	-	-	-	-	-	-	-	-	-	-	-	-	-	+	-	-	-	-	-	-	-	[227]
A & FA	-	-	-	-	-	-	-	-	-	-	-	-	-	-	-	-	-	-	-	-	-	-	-	-	-	-	[228]
A	-	+	-	-	-	-	-	-	-	-	-	-	-	-	-	-	-	-	-	-	-	-	-	-	-	-	[229]
A	-	-	+	-	-	-	-	-	-	-	-	-	-	-	-	-	-	-	-	-	-	-	-	-	-	-	[230]
RA	+	+	-	-	+	-	-	-	-	-	-	-	-	-	-	-	-	-	+	-	-	+	-	-	-	-	[231]
MA	-	+	-	-	+	-	-	-	-	-	-	-	-	-	-	-	-	-	+	-	-	-	-	-	-	-	[232]
A	-	+	-	-	-	-	-	-	-	-	-	-	-	-	-	-	-	-	-	-	-	-	-	-	-	-	[233]
A	-	+	-	-	-	-	-	-	-	-	-	-	-	-	-	-	-	-	-	-	-	-	-	-	-	-	[234]
MA	-	+	-	-	-	-	-	-	-	-	-	-	-	-	-	-	-	-	-	-	-	-	-	-	-	-	[235]
RSA	+	+	-	-	+	-	-	-	-	-	-	-	-	-	-	-	-	-	-	-	-	+	-	-	-	-	[236]
A	+	+	-	-	-	-	-	-	-	-	-	-	-	-	-	-	-	-	-	-	-	-	-	-	-	-	[237]
A	+	-	+	-	-	-	-	-	-	-	-	-	-	-	-	-	-	-	-	-	-	-	-	-	-	-	[238]
A	+	+	-	-	-	-	-	-	-	-	-	-	-	-	-	-	-	-	-	-	-	-	-	-	-	-	[239]
MA	-	+	-	-	+	-	-	-	-	-	-	-	-	-	-	-	-	-	-	-	-	-	-	-	-	-	[240]
GS	-	+	-	-	-	-	-	+	-	-	-	-	-	-	-	+	-	-	-	-	-	-	-	-	-	-	[241]
OPA	-	+	-	-	-	-	-	-	-	-	-	-	-	-	-	-	-	-	-	-	-	-	-	-	-	-	[242]
A	-	-	-	-	-	-	-	-	-	-	-	-	-	-	-	-	-	-	-	-	-	-	-	-	-	-	[243]
CRA	+	-	-	-	-	-	-	-	-	-	-	-	-	-	-	-	-	-	-	-	-	-	-	-	-	-	[244]
A	+	-	-	-	-	-	-	-	-	-	-	-	-	-	-	-	-	-	-	-	-	-	-	-	-	-	[245]
MA	+	+	-	-	+	-	-	-	-	-	-	-	-	-	-	-	-	-	-	-	-	-	-	-	-	-	[246]
A	-	+	-	-	-	-	-	-	-	-	-	-	-	-	-	-	-	-	-	-	-	-	-	-	-	-	[247]
CA	-	+	-	-	-	-	-	-	-	-	-	-	-	-	-	+	-	-	-	-	-	-	-	-	-	-	[248]
MA	-	+	-	-	-	-	-	-	-	+	-	-	-	-	-	-	-	-	-	-	-	-	-	-	-	-	[249]
CBA, BA & MA	-	-	-	-	-	-	-	-	-	-	-	-	-	-	-	-	-	-	-	-	-	+	-	-	-	-	[250]
CA	+	-	-	+	-	-	-	-	-	-	-	-	-	-	-	-	-	-	-	-	-	-	-	-	-	-	[251]
A	+	+	+	-	-	-	-	-	+	-	+	-	-	-	-	-	-	-	-	+	-	-	-	-	-	-	[252]
A	+	+	+	-	-	-	-	-	-	-	-	-	-	-	-	-	-	-	+	-	-	-	-	-	-	-	[253]
A & MA	-	+	-	-	-	-	-	-	-	-	-	-	-	-	-	-	-	-	-	-	-	-	-	-	-	-	[254]
OPA	-	+	-	-	-	-	-	-	-	-	-	-	-	-	-	-	-	-	-	-	-	-	-	-	-	-	[255]
MA & OMA	+	-	+	-	-	+	-	-	-	-	-	-	-	-	-	-	-	+	-	-	-	-	-	-	-	-	[256]
A	-	-	-	-	-	-	-	-	-	-	-	-	-	-	-	-	-	-	-	-	-	-	-	-	-	-	[257]
A	+	-	-	-	-	-	-	-	-	-	-	-	-	-	-	-	+	-	-	-	-	-	-	-	-	-	[258]
A	-	+	-	-	-	-	-	-	-	-	-	-	-	-	-	-	-	-	-	-	-	-	-	-	-	-	[259]
A	+	-	-	-	-	-	-	-	-	-	-	-	-	-	-	-	-	-	-	-	-	-	-	-	-	-	[260]
A	+	+	-	-	+	-	-	-	-	-	-	-	-	-	-	-	-	-	-	-	-	-	-	-	-	-	[261]
A	+	-	-	-	-	-	-	-	-	-	-	-	-	-	-	-	-	-	+	-	-	-	-	-	-	-	[262]

*Abbreviations*: +, performed; -, not performed; A, aorta; AA, afferent arterioles; AT, angiotensin II; BA, basilar artery; BLA, bronchial artery; BRA, bone resistance artery; CA, coronary artery; CBA, cerebral artery; CDA, caudal artery; CRA, carotid artery; D, denuded; ET, endothelin; FA, femoral artery; GA, gonadal artery; GCA, gracilis arterioles; GS, gallbladder strips; IA, iliac artery; IMA, internal mammary artery; MA, mesenteric artery; OMA, omental artery; OPA, ophthalmic artery; PA, pulmonary artery; PSA, prostatic small artery; R, receptor-operated calcium channels; RA, renal artery; RAS, retinal arterioles; RSA, resistance artery; S, store-operated calcium channels; SA, saphenous artery; SE, Sacro/endoplasmic reticulum Ca^2+^-ATPase; SPA, splenic artery; Sr, serotonin; SV, saphenous vein; T, thromboxane A_2_; TA, tail artery; TOV, type of vessel; UA, umbilical artery; V, voltage-operated calcium channels; VA, vertebrobasilar artery.

**Table 2:** General list of antagonists used in studies of signaling mechanism pathways according to their selectivity.

Mechanism Pathways	Antagonists		Ref.
	Selective	Non-Selective	
Endothelial nitric oxide synthase (eNOS)	l-N^G^-NitroArginine-Methyl Ester, l-N^G^-NitroArginine		[268,269,270,271,272,273]
Cyclooxygenase (COX)		Ibuprofen, mefenamic acid, piroxicam, diclofenac, flubiprofen, indomethacin, aspirin	[276,277,278]
Soluble guanylyl cycles (sGC)	1*H*-[1,2,4] oxadiazolo [4,3-*a*]-quinoxalin-1-one	Methylene blue	[279,281,282,283]
Protein kinase G (PKG)	KT 5823	Rp-8-Br-PET-cGMPs	[284]
Adenylyl cyclase (AC)		SQ22536, MDL12330A, 2′5′-dideoxyadenosine	[312]
Protein kinase A (PKA)	Rp-cAMPs, KT 5720, H-89		[313,314,315]
β-adrenergic receptor	Atenolol	Propanolol, nadolol, pindolol	[316]
α-adrenergic receptor	Prazosin (α_1_), RX 821002 (α_2_)		[288,292]
Endothelin receptor	BQ 123 (ET_A_R), BQ 788 (ET_B_R)	Bosentan	[295,296,297,298,299]
Angiotensin II receptor (AT1)	Candesartan cilexetil (ARB & AT_1_), losartan (AT_1_), valsartan (ARB & AT_1_)		[286,287,288,303,304,305,306]
Muscarinic receptor	*N*-methylatropine, tiotropium	Atropine	[300,301,302,318,319]
Thromboxane (TxA_2_)	Ridogrel, ozagrel, furegrelate		[288,292,309]
Serotonin receptor (Sr)	Katanserin (5-HT_2_), WAY 100635 (5-HT_1A_)		[289,290,291,292]
Protein kinase C (PKC)	GF 109203x, staurosporine, Go 6983, chelerythrine, BIM		[286,287,288]
Phosphodiesterase (PDE)	Slidenafil (PDE 5), dipyridamole (PDE 5), zaprinast (PDE 5), T 1032 (PDE 5), rolipram (PDE 4), milrinone (PDE 3)	Papaverine	[152]
Calcium-activated potassium channel (Kca)	Iberiotoxin (BKca), UCL 1684 (BKca), paxilline (BKca), apamin (SKca), TRAM-34 (IKca), clotrimazole (IKca)	Charybdotoxin (BKca & IKca), TEA	[323,327,328]
ATP-sensitive potassium channel (K_ATP_)	Glibenclamide		[330,331]
Voltage-activated potassium channel (K_v_)	XE 991 (K_V_ 7)	4-aminopyridine	[323,332,333]
Inwardly-rectifier potassium channel (K_ir_)		Barium chloride	[334]
Voltage-operated calcium channel (VOCC)	Nifedipine, nicardipine, diltiazem	Verapamil	[335,336]
Store-operated calcium channel (SOCC)	Gandolinium		[340,341]
Inositol triphosphate receptor (IP_3_R)	2-Aminoethoxydiphenyl borate		[337,338,339,340,341]
SERCA	Thapsigargin, cyclopiazonic acid		[342]

Abbreviation: (), selective in receptor.
